# Application of Electrospun Nanofibers for Fabrication of Versatile and Highly Efficient Electrochemical Devices: A Review

**DOI:** 10.3390/polym13111741

**Published:** 2021-05-26

**Authors:** Seyedeh Nooshin Banitaba, Andrea Ehrmann

**Affiliations:** 1 Textile Engineering Department, Amirkabir University of Technology, Tehran 1591634311, Iran; nooshin_bt@yahoo.com; 2 Faculty of Engineering and Mathematics, Bielefeld University of Applied Sciences, 33619 Bielefeld, Germany

**Keywords:** electrolytic cells, batteries, fuel cells, supercapacitors, electrochemical solar cells, sensors

## Abstract

Electrochemical devices convert chemical reactions into electrical energy or, vice versa, electricity into a chemical reaction. While batteries, fuel cells, supercapacitors, solar cells, and sensors belong to the galvanic cells based on the first reaction, electrolytic cells are based on the reversed process and used to decompose chemical compounds by electrolysis. Especially fuel cells, using an electrochemical reaction of hydrogen with an oxidizing agent to produce electricity, and electrolytic cells, e.g., used to split water into hydrogen and oxygen, are of high interest in the ongoing search for production and storage of renewable energies. This review sheds light on recent developments in the area of electrospun electrochemical devices, new materials, techniques, and applications. Starting with a brief introduction into electrospinning, recent research dealing with electrolytic cells, batteries, fuel cells, supercapacitors, electrochemical solar cells, and electrochemical sensors is presented. The paper concentrates on the advantages of electrospun nanofiber mats for these applications which are mostly based on their high specific surface area and the possibility to tailor morphology and material properties during the spinning and post-treatment processes. It is shown that several research areas dealing with electrospun parts of electrochemical devices have already reached a broad state-of-the-art, while other research areas have large space for future investigations.

## 1. Introduction

Electrochemical devices have been part of our lives for a long time. They enable storing energy in batteries [1,2,3], supercapacitors [4,5,6], or fuel cells [7,8,9], can be used as sensors in healthcare and biotechnology [10,11,12], or gain solar energy [13,14,15].

One of the possibilities to increase the efficiency of such electrochemical devices is increasing the contact area between the different parts of the electrochemical devices, typically electrodes and electrolyte, by nanostructuring the electrodes [16,17,18]. Besides diverse physical and chemical methods, such a nanostructure can be achieved unambiguously by the textile technology of electrospinning.

Electrospinning belongs to the primary spinning techniques and enables spinning continuous nanofibers with typical diameters in the range of some ten to some hundred nanometers [19,20,21]. Recently, the needleless and needle-based electrospinning techniques are becoming more and more sophisticated, with modifications of both electrodes as well as with the support of other physical processes, such as Corona-electrospinning or magnetic-field-assisted electrospinning [22,23,24,25].

**Figure 1:**
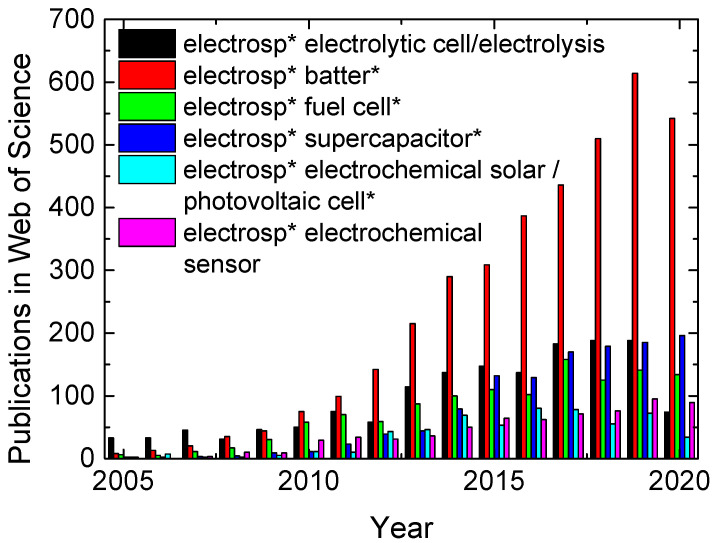
Publications reporting on electrospun nanofiber mats applied in electrochemical cells, found in the Web of Science during the last decades. Data accessed on 18 March 2021.

Typical applications of electrospun nanofiber mats can be found in the areas of biotechnology and biomedicine [26,27,28,29], implant scaffolds [30], micropollutant elimination [31], nanoparticle delivery [32], oil/water separation [33,34], air and water filtration [35,36,37,38], and electrochemical cells [39]. This review gives an overview of the aforementioned kinds of electrochemical cells in which electrospun nanofiber mats are applied. As Figure 1 shows, publications about electrospun nanofiber mats used in these electrochemical cells are increasing from year to year for most sub-topics. Batteries including nanofiber mats are apparently most often investigated, while electrolytic cells based on electrospun nanofiber mats are relatively scarce and most recently did not show increasing numbers anymore. Unexpectedly, in spite of the recent ongoing discussions on fuel cells, supercapacitors have outpaced them in research during the last years. Electrochemical solar cells are only scarcely investigated. Electrochemical sensors, finally, are steadily increasing on a relatively low level. This short overview already shows that electrochemical devices based on nanofiber mats still belong to the topics in which there is much space left for new research approaches, especially in the area of electrolytic cells.

## 2. Electrospinning Process

The electrospinning technique has been identified as a versatile and highly efficient method for fabrication of continuous nanofibers from polymer solution or melt. Generally, an electrospinning set-up contains a high voltage power supplier, a feeding pump, a spinning apparatus (such as spinneret), and a rotational/constant collector. In electrospinning procedure, ultrathin fibers are fabricated in an electrical field created between the collector and spinneret by applying a high voltage. The applied voltage, the distance between the spinneret and collector, the feeding rate, the spinneret inner diameter, and the collector speed belong to the common electrospinning parameters which can influence morphology of the electrospun membranes as well as fiber diameters. Fiber orientation, pore size distribution, and membrane porosity are significant morphology features of the electrospun membranes. Apparently, the morphology characteristics and fiber diameter should be tuned to obtain the most appropriate electrospun fibrous structure for the considered application [40,41,42]. As an example, fiber orientation may lead to production of an electrochemical biosensor with high sensitivity, while it can reduce ionic conductivity of an electrospun electrolyte applicable in lithium ion batteries [43,44].

Optimum electrospinning parameters should be determined for each polymer system as they vary from polymer to polymer. Overall, increasing the applied voltage up to a critical point causes formation of finer fibers due to more stretching of the electrospinning jet resulting from higher repulsion forces in it. On the other hand, thicker or beaded electrospun fibers can be obtained by exceeding the critical voltage as a result of higher velocity as well as smaller size of the Taylor cone [45]. The electrospinning distance should be recognized regarding the evaporation rate and deposition time of the electrospinning jet to gain a uniform membrane. A short electrospinning distance may lead to formation of ribbon-like nanofibers with large diameters, whereas thick fibers are fabricated through a high electrospinning distance [46]. In addition, a critical flow rate is essential to obtain homogenous and beadless electrospun fibers. By increasing the feeding rate beyond the critical point, defective fibers with high diameters in a wide range and large pore sizes may be obtained [47]. Moreover, the receiver type and collector speed mainly influence the fibers’ orientations and so the pore sizes. As the collector speed increases, fibers with higher orientation and less porosity and pore sizes are obtained [48].

Besides the apparatus adjustments, features of the polymer solution or melt influence the membrane morphology and fibers’ diameter. The role of polymer solutions on various features of the electrospun membrane depends on their concentration, viscosity, and conductivity. Enhancement of the solution concentration up to a critical point provides more entanglement between the polymer chains which results in formation of beadless fibers with higher uniformity. Nevertheless, beaded and defective fibers can be obtained beyond the critical point due to drying of the polymer solution on the applied spinneret tip. Notably similar trends have been observed for the viscosity impact on the obtained electrospun fibers by numerous researchers. Furthermore, the electrospinning process highly depends on the Coulomb forces between the electrical field and accumulated charges on the solution surface. Therefore, a polymer solution with very low conductivity cannot be electrospun due to lack of charges which are essential for formation of the Taylor cone. In contrast, polymer solutions with conductivity beyond the critical point cannot also be processed because of spreading of the fibers in the electrospinning environment [49,50].

## 3. Electrolytic Cells

Electrolytic cells use electrical energy to decompose chemical compounds by electrolysis. They can be set up in different forms, e.g., with two-dimensional electrode stacks, spaced without a membrane and connected in a bipolar way [51]. Alternative designs include rotating electrodes to enhance mass transfer without pumping and porous, i.e., three-dimensional electrodes [51]. Possible applications include, e.g., electrochemical CO_2_ reduction [52,53,54], heavy metal removal from wastewater by electrodeposition [55,56,57], nitrogen reduction [58], nitrate removal [59,60] or depolymerization of lignin into renewable aromatic compounds [61], to name just a few.

Due to their large surface-to-volume ration, electrospun nanofiber mats could be used in several of these electrolytic cells. Unexpectedly, this is not fully reflected by the recent literature which shows only few combinations of these two techniques amongst large numbers of papers dealing with one of the topics. Manesh et al. prepared poly(vinylidene fluoride)/poly(aminophenylboronic acid) (PVdF/PAPBA) electrospun composite nanofiber mats to be inserted into electrolytic cells; however, these were used to detect glucose, i.e., they worked as sensors and not as electrodes [62]. Many papers report about using an electrolytic cell to measure the conductivity of the spinning solution (e.g., [63,64,65]) or apply an electrolytic cell to perform electrochemical deposition on a nanofiber mat [66,67]. As most of the authors mention, the fiber and mat morphologies play an important role for this application. Usually, finer nanofibers, providing a higher specific surface, are preferred [62,63]. In addition, some electrospinning processes allow for producing nanofibers with pores or even with additional dendrite-like structures on the fiber surface [64] which further increases the specific surface and thus the contact area to the surrounding material.

**Figure 2:**
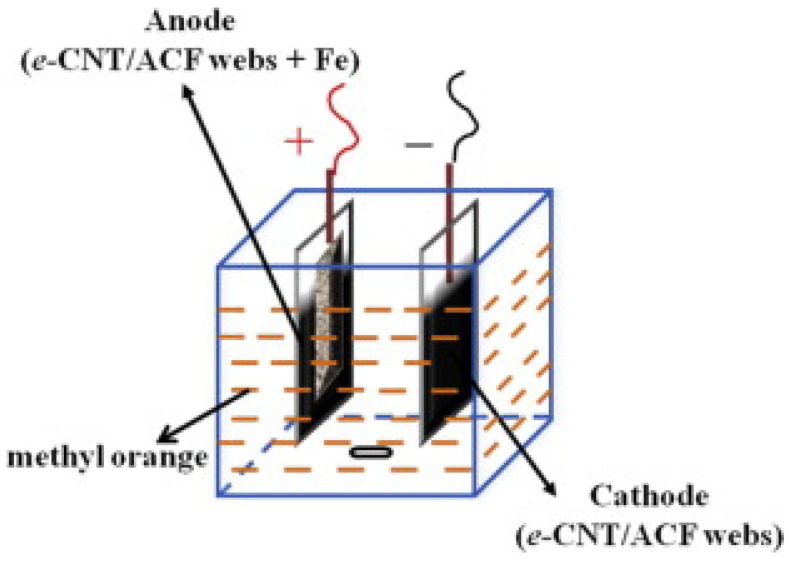
Scheme of the electrolytic cell used for methyl orange degradation. Reprinted from [68], with permission from Elsevier.

Electrolysis based on electrospun nanofibers mats can, e.g., be found in some dye-degradation applications. Sun et al., for example, used activated carbon nanofibers modified with carbon nanotubes (CNTs) as electrodes for the electrochemical degradation of methyl orange (Figure 2) [68]. Li et al. prepared poly(acrylonitrile) (PAN) and Fe/PAN electrodes by electrospinning and reported an increase of the electrochemical degradation due to the higher specific surface area (SSA) and higher amount of mesopores [69]. Hwang et al. showed that carbon nanofibers, prepared by electrospinning followed by stabilization and carbonization, supported the toluene removal efficiency by electrolysis when used as cathodes or anodes [70].

Degradation of organic particles was examined by Kim et al., using bisphenol A as a model substance. They prepared Sb-doped SnO_2_ nanofibers by electrospinning SnCl_2_ and SbCl_3_ with polyvinylpyrrolidone (PVP) dissolved in a dimethylformamide (DMF) solution, partly with a shell containing RuO_2_, followed by calcination of the polymer. These materials were investigated as anodic materials for anodic oxidation of bisphenol A and found highly suitable for this application, with an eleven-fold faster organic oxidation than the reference of pure RuO_2_ nanofibers combined with low noble metal content [71].

Recently, Chen et al. used electrospun polyethylene oxide (PEO)/ionomer/graphene oxide (GO) nanofiber mats for water dissociation and found high stability under repeated cycling, even for high current densities. These findings let the authors suggest such three-dimensional bipolar membranes for the application in CO_2_ electrolysis devices where high current densities are applied [72].

The electrocatalytic activity of Pt-IrO_2_ electrospun nanofibers in water electrolysis was investigated by Wang et al. [73]. They found that, for different ratios of Pt to Ir, these nanofiber mats showed better catalytic performance than commercial IrO_2_ catalysts and at the same time reduced the amount of expensive Ir.

Mugheri et al. used NiO nanostructures deposited on MoS_2_ nanofiber mats for water splitting. For electrospinning, the NiO nanostructures were inserted together with ammonium phosphomolybdate hydrate and PVP into a spinning solution. The nanofiber mat was afterwards calcinated at 500 °C to remove the polymer. This electrocatalyst nanofiber mat showed good stability, durability, and high efficiency in the hydrogen evolution reaction [74].

Similarly, diverse other materials were electrospun by using different polymers as spinning agents and different metals to form the final nanofiber mat after calcination, and applied for electrocatalytic water splitting, i.e., hydrogen or oxygen evolution reaction. Some of these metals or metal oxides are Co_3_O_4_ [75], Co/Mo_2_C [76], and Ni_3_V_2_O_8_ nanocube decorated nanofibers [77].

Other applications of nanofiber mats in electrolytic cells are, e.g., disinfection of water from bacteria by combining electrolysis with physical filtering through a PAN/polyurethane (PU)/polyaniline(PAni) nanofiber mat with embedded single-walled carbon nanotubes [78]; elimination of urea from wastewater by electrospun Ni/C nanofiber mats [79]; and N_2_ fixation to NH_3_ by the electrocatalytic nitrogen reduction reaction, e.g., by carbon nanofibers with embedded MnO nanocrystals [80].

As these examples show, electrospun nanofiber mats are mostly applied in electrocatalytic water splitting and degradation of dyes and other contaminants. This recent restriction on a relatively small range of research topics within the field of electrolytic cells suggests broadening the possible range of applications of nanofiber mats from diverse materials by further research in neighboring regions.

## 4. Batteries

Rechargeable or secondary batteries are electrochemical power sources commonly utilized in portable devices such as camcorders, mobile phones, laptops, and electric transportations. In general, batteries are comprised of one or more electrochemical cells. Positive electrode (cathode), negative electrode (anode), porous separator membrane, and ionic conductive electrolyte are the essential components for fabrication of each electrochemical cell. Lead-acid, nickel cadmium, and lithium ion belong to the well-known secondary batteries. However, lithium ion batteries (LIBs) have shown superior advantages compared with the other rechargeable ones. High specific energy density as well as small size and low mass are outstanding and distinctive characteristics of the LIBs [81,82].

**Figure 3:**
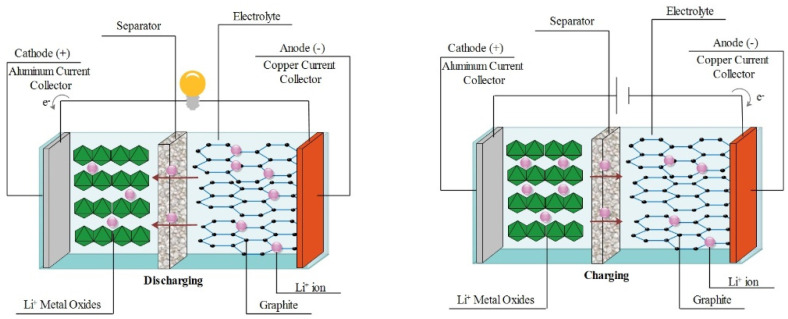
Schematic showing the intercalation mechanism in Li-ion batteries through charge and discharge. Reprinted from [85], with permission from Elsevier.

Increasing progress in technology has forced researchers to design batteries with higher energy density, lighter weight, and more flexible structure. A basic LIB comprises of a lithium metal oxide electrode as cathode, a graphite-based anode, a porous polypropylene (PP) or polyethylene (PE) film as separator, and a lithium salt/solvent solution as electrolyte (Figure 3). Charge/discharge procedure of the LIBs is performed through chemical reactions. During the charge process, free lithium ions migrate from the cathode toward the anode, via diffusion into the ionic conductive electrolyte. Simultaneously, the electrons travel toward the anode through the external circuit and form LiC_6_ compound in the anode material. Apparently, the reverse behavior takes place in the discharge step [81,82]. Over the past decades, most research in advanced development of LIBs has emphasized the use of electrospun fibers for fabrication of versatile and highly efficient components [83,84]. Recent progresses in the fabrication of electrospun cathode, anode, separator, and electrolyte are provided in the following section.

### 4.1. Electrospun Cathode Materials

Electrochemical performance of the batteries, such as potential window and storage capacity, is mostly affected by the cathode material. In fact, the number of extracted lithium ions from the cathode electrode determines the battery capacity. The energy could be stored in the cathode materials through two various conversion and intercalation techniques. In the conversion mechanism, lithium insertion and extraction are associated with changes in the crystalline structure of the applied cathode material, while the cathode structure acts as a host in the intercalation mechanism. So, the lithiation/delithiation can reversibly occur in the intercalation cathode materials. Notably, low electron conduction as well as high volume expansion have been reported as challenges linked with the conversion cathode materials. Therefore, the intercalation cathode structures have received more attention from numerous researchers. Among various types of intercalation structures (transition metal oxides, chalcogenides, and poly anions), transition metal oxides and poly-anionic compounds have displayed superior characteristics such as higher energy storage and greater operating voltage, while development of chalcogenide materials has been influenced by their irreversible structure [86,87].

Layered structures have been identified as the most widely applied electrode materials in the commercial LIBs. They are commonly presented by the chemical formula of LiXO_2_, where X could be Co, Mn, or Ni. This group of cathode materials was first introduced by discovering of LiCoO_2_ in the 1990s. During discharging, LiCoO_2_ hexagonal cells are formed in layered structures, whereas Li_1−x_CoO_2_ monoclinic phases are created as a result of Li^+^ ion removal during the charging procedure. The structural instability of LiCoO_2_ cathode material is a major drawback associated with these materials which has resulted in lower practical capacity (140 mAh·g^−1^) compared with the reported theoretical capacity (280 mAh·g^−1^). So, LiNiO_2_ was introduced to address the low experimental storage capacity. However, poor thermal stability, along with low electrochemical activity restricts practical usage of the layered LiNiO_2_. Therefore, LiNi_0.5_Mn_0.5_O_2_ was presented by Ohzuku et al. as a modified compound. Based on various analyses, this cathode material has revealed appropriate structural stability due to the existence of Mn^4+^ cations. In addition, it has represented a stable structure up to 300 °C, which is a crucial function for being applied in commercial batteries. Moreover, it has shown superior storage capacity (200 mAh·g^−1^) compared with the LiCoO_2_ layered structure, although limitation of Li^+^ ion extraction due to presence of Ni in the cathode material has caused synthesis and evaluation of LiCo_x_Ni_y_Mn_1−x−y_O_2_ ternary compound materials such as LiCo_1/3_Ni_1/3_Mn_1/3_O_2_ [85,88].

Spinel oxides with the general chemical formula of LiM_2_O_4_ (e.g., LiMn_2_O_4_) are another group of cathode materials. Compared with layered structures, they are safer and more affordable. Three-dimensional paths in such structures facilitate Li^+^ ion diffusion and therefore enhance the rate capability. Nevertheless, capacity fading is a critical disadvantage associated with these materials. To overcome the aforementioned obstacle, doping mechanism has been widely reported to reduce Jahn-Teller active Mn^3+^ ions and thus to enhance the electrochemical characteristics. Mg, Ni, Cr, Al, and many more metal elements belong to the applied dopant materials. As an example, LiNi_0.5_Mn_1.5_O_4_ compound has illustrated superior rate capability and wider potential window compared with LiMn_2_O_4_ spinel oxide structure [89,90].

Poly-anionic compounds, with the general chemical structure of (XO_4_)^3−^ (X = P, Si, S, etc.), have also received much attention as cathode material of Li-ion batteries. LiFePO_4_ and LiMnO_4_ are of the well-known poly-anionic cathode materials. This could be linked with their great power capability and proper structural stability. Nevertheless, low conductivity of the aforementioned materials has restricted their applications [91,92].

Synthesis of highly efficient cathode materials is considered as a key building block toward progress of energy storage systems with high power and proper capacity in the future. Numerous researchers have illustrated great potential of the electrospun structures as cathode material of the Li-ion batteries. Besides storage capacity, cycling stability is considered as an important parameter for determination of the efficiency and capability of a designed cathode material. Cycling durability is measured by calculation of the storage capacity in various cycles. Apparently, a more ideal battery structure would be obtained through increment of the cycling stability. Commercial LIBs normally show cycling stability during first 300 to 500 cycles (or about 2 to 3 years) [93,94]. As an example, electrospun LiCoO_2_ fibers (148 mAh·g^−1^) result in higher cyclic stability compared with the LiCoO_2_ powders (138 mAh·g^−1^) [95]. Notably, the enhanced capacity of the nanofibrous structures could also be further improved through modification of the electrospun fibers by coating methods. In addition, electrospun LiM_2_O_4_ structures provide faster diffusion of the lithium ions as well as the promoted cycling stability. In fact, the highly porous structure of the electrospun fibers leads to reduction of the degradation rate during charging/discharging processes [96,97]. Moreover, presence of the carbon nanofibers in the poly-anionic compounds such as LiFePO_4_ compensates the poor ionic conductivity of this category of cathode materials and causes approaching a more appropriate rate capability [98]. The most recent approaches in fabrication of electrospun fibers applicable as cathode materials are summarized in Table 1.

### 4.2. Electrospun Anode Materials

Intercalation-, conversion-reaction-, and alloying-reaction-based materials are various categories which have been applied as anodes of LIBs. In the intercalation group, the Li^+^ ions are placed between the layers of the utilized anode material. Graphite is the most well-known intercalation-based anode structure. In the low voltage range (<0.25 V), high capacity of 360 mAh·g^−1^ along with 100% discharge/charge efficiency have been recorded for this anode structure, whereas most of the electrolyte solvents (e.g., ethylene carbonate (EC), propylene carbonate (PC), and so on) are decomposed between 0.5 and 0.7 V, resulting in the formation of a solid–electrolyte interface (SEI) layer. It is worth noting that proper ionic conductivity, low electrical conductivity, and great stability are the essential characteristics of the ideal SEI layer. Overall, poor capacity is a major drawback associated with the graphite anode materials [105,106].

Surface-to-volume ratio enhancement of the applied anode material is an effective method toward providing more accommodations for the Li^+^ ions. Therefore, various studies have been devoted to fabrication of carbonaceous nanomaterials. PAN is the most common precursor for synthesis of electrospun carbon fibers. This could be linked with the simple fabrication procedure, proper mechanical characteristics, and affordable cost. However, environmental concerns associated with DMF, an essential solvent for dissolving PAN polymer, has led to investigation for new precursor resources including lignin, polyvinyl alcohol (PVA), and many more [105,106]. Kim et al. [107] reported a large capacity of 450 mAh·g^−1^ derived from the electrospun PAN nanofibers. In another attempt, Chen et al. [108] introduced a large capacity of 1150 mAh·g^−1^ at 0.1 A·g^−1^ for a hollow CNT/CNF composite. In addition, Culebras et al. [109] claimed a high capacity of 611 mAh·g^−1^ after 500 cycles for a CNF mat obtained from lignin/polylactic acid (PLA) precursor. Moreover, Nan et al. [110] revealed a large capacity of 841 mAh·g^−1^ for a carbon nanofiber membrane synthesized from a PVA precursor. Notably, fabrication of porous and hollow CNFs could result in enhancement of the discharge capacity through increment of the Li^+^ ions’ spaces. Further, it causes easier interaction between electrode and electrolyte components by reduction of the distances between ions and electronics [111].

Conversion-reaction-based anode materials work based on the faradic reaction. Metal oxides (e.g., Co_3_O_4_, Cu_2_O, etc.), metal nitrides (M_x_N_y_, where M is Ni, Fe, Mo, etc.), metal sulfides (M_x_S_y_, where M is Ni, Fe, Mo, etc.), and metal phosphides (Li_x_M_y_P_4_, where M is V, Cu, Ti, etc.) are of the conversion-reaction-based structures. These kinds of anode structures are able to provide capacity in the range from 350 mAh·g^−1^ (Cu_2_S) to 1800 mAh·g^−1^ (MnP_4_). Despite the high capacity, the conversion-reaction-based materials suffer from low potential, poor cycling durability, and high volume changes during extraction and insertion of the Li^+^ ions. In order to suppress volume changes of the conversion-reaction-based anode structures, fabrication of porous nanomaterials has received wide attention. Existence of pores in such materials manages the volume changes during lithiation/dilithiation processes through providing sufficient spaces for extraction and contraction of the applied anode material. In addition, combination of these materials with the carbonaceous structures has been claimed as another effective method for control of the volume changes [105,106]. Zhang et al. [112] reported a large capacity of 835 mAh·g^−1^ at 0.2 A·g^−1^ after 100 cycles for a Mn_3_O_4_/CNF composite membrane. In addition, a CoO/CNF three-dimensional mat revealed large discharge capacity of 853.5 mAh·g^−1^ after 100 cycles [113]. Moreover, electrospun NiO fibers have exhibited a discharge capacity of 784 mAh·g^−1^ at 0.08 A·g^−1^ [114].

Alloying-reaction-based materials have been considered as the third category of anode structures. Various metals which are able to be alloyed with lithium (such as Se, S, etc.) are classified in this group. During the charging procedure, lithium ions make an alloy with the applied alloying-reaction-based structures. The aforementioned class of anode structures could reveal various capacities based on the applied alloying metal ranging from 660 mAh·g^−1^ (Sb) to 4200 mAh·g^−1^ (Si). The major drawback linked with this materials are volume changes during insertion and extraction of the lithium ions, which could be suppressed by size reduction of the applied particles as well as combination with the carbonaceous materials [105,106]. Jang et al. [115] revealed a discharge capacity of 560 mAh·g^−1^ after 80 cycles for an electrospun Co-Sn/CNF composite. In addition, a high discharge capacity of 830 mAh·g^−1^ at 0.4 A·g^−1^ after 100 cycles was claimed for the Si/CNF three dimensional structure [116]. Furthermore, a SnS/CNF composite membrane presented 648 mAh·g^−1^ discharge capacity at 0.2 A·g^−1^ after 500 cycles [117]. Table 2 describes some of the most recent advancements carried out for the fabrication of highly efficient electrospun anodes.

### 4.3. Electrospun Separator

The separator is another essential key component of LIBs. Prevention of the contact between positive and negative electrodes and transportation of the Li^+^ ions between the electrodes, along with retaining the liquid electrolyte are apparent responsibilities of this crucial element. Regarding the role of a separator part in LIBs, an ideal separator must provide sufficient ionic conductivity, wettability, and permeability. In addition, dimensional, thermal, and electrochemical stabilities are other vital characteristics of an appropriate separator. Porous PP or PE membranes are common structures utilized as separators in LIBs. However, poor conductivity as well as low wettability are the most well-known downsides associated with these kinds of separators. Among various techniques applied for the fabrication of ideal separators, electrospun membranes have revealed more appealing features. The highly porous structure of the nanofibrous mats, interconnected pores, and large surface-to-volume ratios of the electrospun fibers provide proper wettability and permeability for separators [128,129].

The electrospun separators are mainly divided into four classes, including: monolayer, multilayer, modified, and composite membranes. Monolayer separators are mainly fabricated from one polymeric precursor such as PVDF [130], polyimide (PI) [131,132], PAN [133,134], and so on, while multilayer membranes are obtained by sequential fabrication of various polymer precursors. In this method, appropriate advantages of the various polymers (such as thermal stability, dimensional stability, electrochemical performance, etc.) could be attained in one separator membrane. PVDF/poly(m-phenylene isophthalamide) (PMIA) [135], PVDF/polyethylene terephthalate (PET) [136], PVDF/PI [137], and polysulfonamide (PSA)/PET [138] are some of the reported multilayer electrospun separators. Post-treatment of the electrospun fibrous membranes is a great technique for modification and improvement of various characteristics. Dip-coating [139,140], in situ polymerization [141,142], and atomic layer deposition [143] are significant modification methods. In such procedures, a material is introduced into the electrospun separator, which results in improvement of its final properties. Direct electrospinning of the combination of two polymer solutions (e.g., PAN/PU [144], PAN/Lignin [145], PSA/PVDF-HFP [146], and so on) or filler-loaded polymer solution (e.g., PAN/SiO_2_ [147], PI/Al_2_O_3_ [148], Nylon6,6/TiO_2_ [149], etc.) leads to the fabrication of composite separator membranes with enhanced hydrophilicity and heightened thermal stability. Besides the role of the polymer type, the morphology of the electrospun fibers also influences the obtained electrochemical behavior. As an example, fabrication of finer fibers results in increment of electrolyte uptake and so enhancement of the ionic conductivity. Therefore, the morphology of the electrospun fibers should be tuned to approach appropriate electrospun separators with proper electrochemical characteristics [150]. A summary of the recent progresses in the fabrication of electrospun separators is provided in Table 3.

### 4.4. Electrospun Electrolyte

Cycle life, power density, and safety of LIBs are influenced by their electrolyte elements. In batteries, the electrolyte component transports the Li^+^ ions between the electrodes to complete the charge and discharge cycles. In the commercial LIBs, liquid electrolytes, consisting of an organic solvent and a lithium salt, are mainly utilized to fabricate the electrochemical cells. However, flammability of the applied solvents requires metallic sealing for the battery, which results in the production of heavy, inflexible, and expensive cells. Solvent-free electrolytes have been widely recommended as a solution toward fabrication of lightweight, safe, and cost-effective batteries. In such electrochemical cells, the solid electrolyte structure supports the role of both electrolyte and separator. In fact, it prevents the contact between electrodes and transports the Li^+^ ions between them. All-solid-state electrolytes are generally synthesized based upon polymeric structures and inorganic solid materials [160,161].

Polymeric solvent-free electrolytes are synthesized based on dispersion of a lithium salt (LiBF_4_, LiClO_4_, LiTFSI, etc.) in a polymer matrix (e.g., PAN, PVDF, PEO, poly(methylmethacrylate) (PMMA), etc.). They are mainly fabricated in the formation of casted films. Poor ionic conductivity has been claimed as the main drawback of the polymeric electrolytes. Formation of polymer/salt crystalline phases has been introduced as one of the inhibitor parameters for Li^+^ ion movements during cycling processes. In such combinations, Li^+^ ions are transported between the electrodes through polymer chain local motions or hopping mechanism. So, reduction of the glass transition temperature as well as increment of the amorphous regions are key solutions for enhancement of the ionic conductivity of the polymer films [161,162]. Therefore, introduction of particulate fillers (such as SiO_2_ [163], Al_2_O_3_ [164], TiO_2_ [165], and many more) and plasticizer molecules (such as EC, PC, etc.) into the polymer matrix have been reported as influential methods for enhancement of the ionic conductivity. Particulate fillers placed between polymer chains of the utilized polymer matrix cause reduction of the crystalline phases. Thus, the polymer chains would be able to move easily and so accelerate transportation of the Li^+^ ions. In addition, inserted fillers enhance ion pair dissociation of the applied lithium salts, which obviously influences the ionic conductivity [163,164,165]. In 2017, Freitag et al. reported higher conductivity of the electrospun solvent-free electrolytes in comparison with that of the casted ones. Based on this research, PEO/SN/LiBF_4_ electrospun electrolyte could exhibit a high ionic conductivity of 0.2 mS·cm^−1^ [166]. In a similar research, they showed a high ionic conductivity of 0.1 mS·cm^−1^ for the electrospun PEO/SN/NaBF_4_ membrane [167]. Higher ionic conduction of the solvent-free electrospun structures compared with that of the solution-casted membranes are linked with two main issues. First, small pores between the electrospun fibers are excellent pathways for transportation of the Li^+^ ions. Second, fast evaporation of the solvent during electrospinning procedure does not allow the polymer chains and lithium salts to form polymer/salt crystalline regions. So, concentration of the free lithium ions increases in the electrospun membranes, leading to enhancement of the ionic conductivity [43,168]. It is worth noting that electrochemical behavior of the electrospun mats highly depends on the morphology of the fabricated fibers. Based on the obtained results, ionic conductivity could be enhanced by reduction of the average fiber diameter to an optimum range. This may be linked with formation of tiny pores and so more ideal pathways for fast transportation of the Li^+^ ions. Nevertheless, further decrement in average diameter of the fabricated fibers could result in reduction of the ionic conductivity. This trend is attributed to formation of more crystalline regions in the structure of finer fibers as well as superior density of the electrospun fibrous mats containing thinner fibers [43,169]. A comparison between ionic conductivity of the electrospun and solution-casted electrolytes with similar chemical compositions is provided in Table 4.

**Figure 4:**
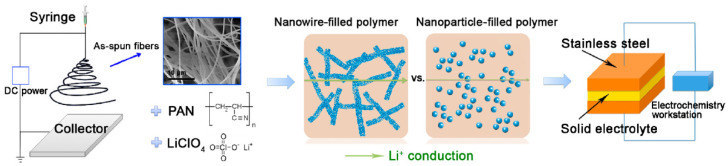
Schematic illustration of the synthesis of ceramic nanowire-filled polymer-based composite electrolytes, together with the comparison of possible lithium-ion conduction pathway in nanowire-filled and nanoparticle-filled composite electrolytes, and illustration of the electrode. Reprinted with permission from [174]. Copyright (2015) American Chemical Society.

Inorganic solid materials have also been evaluated as applicable all-solid-state electrolyte in the LIB structures. Crystalline structure of such materials facilitates fast migration of the Li^+^ ions between the positive and negative electrodes. Garnet-type, LISICON-like, NASICON-like, and Argyrodite are well-known inorganic solid structures which are able to reveal high ionic conductivity as high as the liquid electrolytes. As an example, lithium germanium phosphorous sulfide (Li_10_GeP_2_S_12_), classified in the LISICON-like category, has shown a high ionic conductivity of 10 mS·cm^−1^ at room temperature. Nevertheless, the existence of rare and expensive elements in the structure of inorganic solid materials, along with low flexibility, has restricted their practical usage. To overcome the aforementioned obstacles, several researchers have suggested applying electrospun inorganic solid materials as fillers in the polymeric membranes [173,174,175]. So, a high ionic conductivity of 0.25 mS·cm^−1^ has been reported for a PEO-based polymeric membrane incorporated with the electrospun Li_6.4_La_3_Zr_2_Al_0.2_O_12_ fillers [173]. In addition, ionic conductivity of a PAN-based casted film was enhanced up to 0.24 mS·cm^−1^ through introduction of Li_0.33_La_0.557_TiO_3_ nanofibers (Figure 4) [174]. Moreover, Liu et al. [175] have reported that dispersion of well-oriented ceramic nanowires instead of random nanowires in a host polymer matrix could cause more ionic conductivity resulting from faster transportation of the Li^+^ ions. Hence, morphological features play a key role to obtain ideal electrochemical nanofibrous components.

So far, lithium secondary batteries have been widely utilized as energy storage devices in various applications. Regarding progress and development of technology, production of storage power tools with superior efficiency and improved function has been crucial. In order to achieve this significant aim, all battery components, including anode, cathode, separator, and electrolyte should be enhanced and augmented. In recent decades, the electrospinning technique has shown a great potential to approach versatile and highly efficient fibrous structures for designing advanced LIBs. Despite the reported advantages of electrospun components of LIBs, several drawbacks, such as poor mechanical strength, low electrical conductivity, and poor ionic conduction, have restricted their practical applications. Meanwhile, such downsides could be eliminated through further evaluation and modification of electrospun membranes. By addressing the mentioned challenges, fabrication of all-solid-state electrospun batteries comprising of nanofibous electrodes along with nanofibrous electrolyte could be considered as the main trend in the near future.

## 5. Fuel Cells

Fuel cells are based on electrochemical reactions with an external source for the reacting material [176,177]. They have efficiencies around 40–85%, which is higher than those of turbine generators or a diesel engine and a capacity range comparable with photovoltaics or a turbine generator, making them highly interesting for rural areas with limited access to the public grid or for uninterruptible power supplies [177].

Technologically, they work by a reversed electrolysis reaction, creating electricity and heat by the reaction of oxygen and hydrogen to water. A fuel cell consists in principle of two electrodes, i.e., cathode and anode, separated by an electrolyte, and the external electric circuit used to gain energy from the cell. Depending on the kind of electrolyte, fuel cells are separated into alkaline, phosphoric acid, solid oxide, molten carbonate, direct methanol, and proton exchange fuel cells [178].

Similar to the aforementioned batteries, fuel cells can contain electrospun nanofiber mats for different purposes which will be presented in this section.

### 5.1. Electrospun Cathode Materials

Diverse studies investigated possibilities to prepare cathodes for fuel cells from electrospun nanofiber mats.

**Figure 5:**
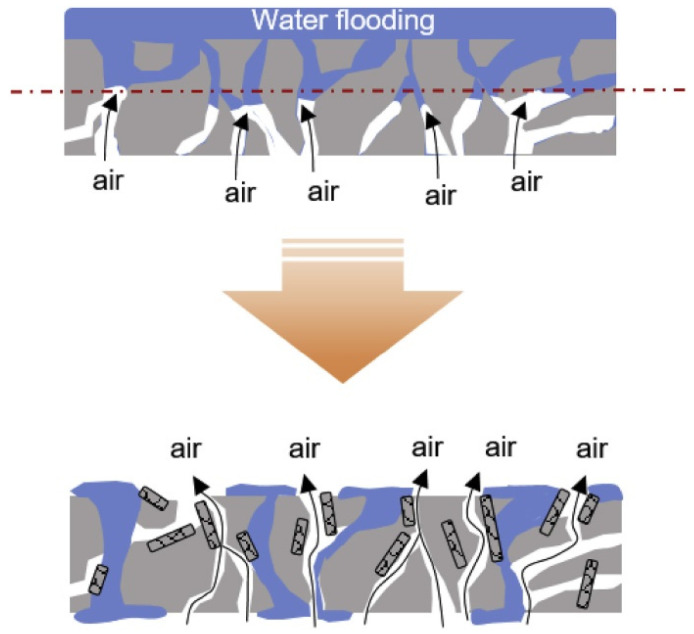
Improved water management due to graphitized carbon nanofibers incorporated in the cathode layer. Reprinted from [180], with permission from Elsevier.

In proton exchange fuel cells, for example, the problem occurs that the water generated at the cathode is not properly transported at high current densities, resulting in so-called water flooding, and can in addition support corrosion of the carbon electrode, in this way reducing also the long-term stability [179]. To reduce this problem, Chung et al. suggested introducing hydrophobic graphitized carbon nanofibers, achieved by annealing electrospun PAN nanofibers up to temperatures of 1000–2500 °C, into the cathode layer, resulting in water-free regions in the electrode, as depicted in Figure 5 [180]. They found highest peak power densities for a concentration of 45 wt.% graphitized carbon nanofibers by reducing water flooding [180]. Slack et al. suggested PVDF as a binder for Pt/C cathodes to reduce carbon corrosion accelerated stress [181]. They compared electrospun nanofiber cathodes with Nafion/PVDF and Nafion/poly(acrylic acid) (PAA) binders with a slurry cathode with neat Nafion and Nafion/PVDF binder and found that the presence of the hydrophobic PVDF reduced carbon loss and increased the binder strength [181]. In an earlier investigation, the group studied PtCo/C and Pt/C catalyst powders integrated in electrospun nanofibrous mats in comparison with conventional sprayed cathode membranes, again using Nafion/PAA as a binder, and found a higher initial performance as well as a superior long-term stability for the electrospun cathodes [182]. Similarly, Khandavalli et al. used PAA to reduce agglomerations of platinum on carbon catalyst particles in catalyst inks [183]. In another earlier work, Zhang et al. showed the advantages of electrospun nanofiber cathodes under low and high feed gas humidification with similar fiber composition [184]. Wei et al. used graphene-doped electrospun nanofiber mats for both cathode and anode and found high conductivity and great porosity of these electrodes, making them well suitable for fuel cells after Pt loading [185].

Cathodes in direct methanol fuel cells can also be prepared by electrospinning. Membranes used in these fuel cells must combine high proton conductivity with low methanol permeability to avoid fuel from anode reaching the cathode, which is a problem for the typically used Nafion membranes [186]. Liu et al. compared sulfonated poly(ether ether ketone) (SPEEK) membranes, Nafion membranes, and SPEEK membranes doped with sulfonated carbon nanofibers (SCNFs) [187]. They found increased mechanical strength, proton conductivity, and decreased methanol permeability for SPEEK/SCNF composites, as compared to the other membranes. In a microfluidic fuel cell, working on formic acid as fuel and KMnO_4_ as oxidant, Jindal et al. applied a CN_x_ nanofiber mat as cathode catalyst and found a power density similar to gold or platinum catalysts [188]. Electrospinning of CN_x_ nanofibers was performed by producing CN_x_ nanoparticles and spinning them with PAN, Nafion and carbon black powder from a DMF solution. In this way, usually a CN_x_ layer on PAN nanofiber was produced, bound by the Nafion dispersion, while sometimes nodes in the PAN nanofibers stemming from CN_x_ nanoparticles supported on carbon black were found [189]. Using an electrospun Fe-N/C nanofiber mat as catalyst for a direct methanol fuel cell, Mei et al. found the multi-scaled porous structure to be beneficial for the cathode catalyst in a fuel cell [190] They concluded that while micropores helped accommodating active sites, the meso- and macropores supported oxygen supply to the active surfaces. In their study, high oxygen reduction reaction was found in acid media, combined with good stability and methanol tolerance.

**Figure 6:**
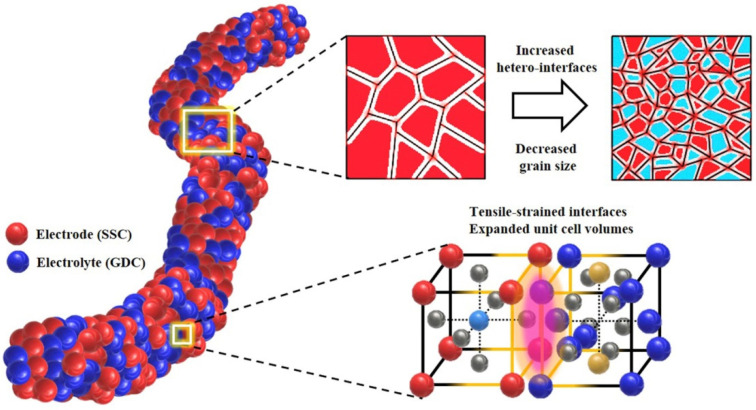
Schematic of electrode/electrolyte nanofiber from Sm_0.5_Sr_0.5_CoO_3−δ_ (SSC) and Gd_0.2_Ce_0.8_O_1.9_ (GDC) with increased hetero-interface, decreased grain size, and expanded unit cell volumes. Reprinted from [195], with permission from Elsevier.

While molten carbonate fuel cells scarcely contain electrospun nanofibrous cathodes, solid oxide fuel cells use them often. One of the problems of solid oxide fuel cells is the high operating temperature of typically 800 °C and higher when preparing solid oxide fuel cells based on an electrolyte from yttria-stabilized zirconia, resulting in relatively low lifetimes and high costs. To solve this problem, Zhi et al. suggest using a 3D nanofiber network as the cathode to reduce operation temperature to 750 °C [191]. They used an electrospinning solution of PAN in DMF, blended with La_0.58_Sr_0.4_Co_0.2_Fe_0.8_O_3_, and heat treated the electrospun nanofibers at 800 °C to reach nanofibers with a perovskite structure. Further improvement was obtained by adding gadolinia-doped ceria into the 3D nanoporous network. In an earlier study, the group investigated yttria-stabilized zirconia nanofiber networks infiltrated with La_0.8_Sr_0.2_MnO_3_ and found reduced polarization resistance in comparison to bulk cathodes from the same materials [192]. Enrico et al. used water instead of DMF for electrospinning a sol–gel solution to prepare La_0.6_Sr_0.4_Co_0.2_Fe_0.8_O_3−δ_ nanofibers [193]. After heat treatment of the nanofibers, they were applied on a Ce_0.9_Gd_0.1_O_1.95_ electrolyte disk to prepare a symmetrical fuel cell which was investigated at temperatures of 550–950 °C and found to have low performance reduction for operation at 750 °C. A lower working temperature of 650 °C was achieved by using a composite cathode from electrospun La_0.8_Sr_0.2_Co_0.2_Fe_0.8_O_3−*δ*_ nanotubes/Ce_0.8_Gd_0.2_O_1.9_ [194]. Different materials were suggested by Ahn et al. who prepared Sm_0.5_Sr_0.5_CoO_3−δ_ and Gd_0.2_Ce_0.8_O_1.9_ composite nanofibers by electrospinning and found a significant increase of the electrode performance as compared to pure Sm_0.5_Sr_0·5_CoO_3−δ_ cathodes [195]. The precursor-based one-step electrospinning process was found to be advantageous to prepare increased grain boundary density and maximum hetero-interfaces between both phases (Figure 6), which supports oxygen reduction reaction at the cathode and thus an increased fuel cell performance.

Phosphoric acid fuel cells belong to the most often used ones. Here, the electrolyte mainly contains phosphoric acid (H_3_PO_4_), a proton conductor delivering protons from the anode to the cathode [196]. In phosphoric acid fuel cells, again electrospun nanofiber mats can be applied as cathodes. Skupov et al., for example, modified cathodes for medium-temperature phosphoric fuel cells based on polybenzimidazole membranes [197]. They prepared PAN nanofiber mats from DMF, blended with carbon black and partly PVP, stabilized them in air and subsequently carbonized them at 900–1100 °C in vacuum. Afterwards, the nanofiber mats were partly loaded with Pt or Ni. These cathodes were found to show improved polarization values and increased catalytic activity due to the increased specific surface of the more porous nanofibers. Previously, the same group reported on gas diffusion electrodes for fuel cells, prepared by sequential oxidation and pyrolysis of electrospun nanofiber mats prepared from PAN, decorated with Pt [198]. Besides these few examples, phosphoric acid fuel cells are normally not prepared with nanofibrous cathodes.

Similarly, there are only few reports available on nanofibrous cathodes for alkaline fuel cells which use typically potassium hydroxide in water or nowadays an alkaline polymer membrane as the electrolyte. As an example, Uhm et al. prepared CNFs with embedded non-precious metals for the oxygen reduction reaction in alkaline ethanol fuel cells by electrospinning and subsequent carbonization [199]. They found that the Fe and Co metals supported nitrogen and oxygen incorporation on the CNF surface instead of directly being part of the oxygen reduction reaction.

Besides the possible applications of electrospun nanofiber mats as cathodes in fuel cells, the next sub-section gives a brief overview of applying nanofibrous materials as anodes.

### 5.2. Electrospun Anode Materials

**Figure 7:**
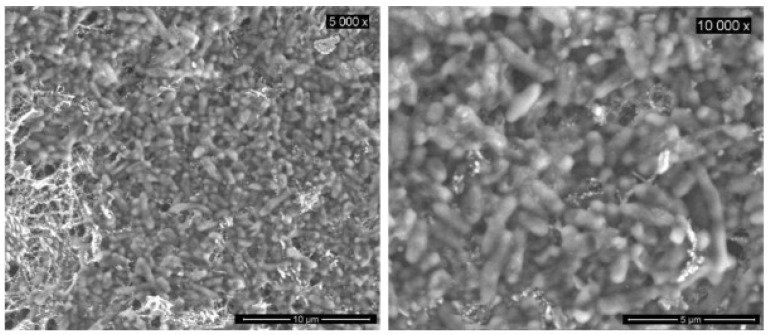
Scanning electron microscope images of electro-activated *Escherichia coli* K12. Reprinted from [202], with permission from Elsevier.

An interesting application of nanofiber mats used as anodes in fuel cells is given by microbial fuel cells in which the anodes mostly define the fuel cell performance [200,201]. These cells generate electricity by oxidizing biodegradable organic matter, such as glucose or proteins, in the presence of microorganisms [202]. Garcia-Gomez et al. studied TiO_2_–C/C nanofiber mats and found good electrical performance, combined with the ability to host a dense biofilm of electro-activated *Escherichia coli* (Figure 7), which could be used for the bioconversion to electricity in a microbial fuel cell [202]. The nanofiber mats used in their experiments were prepared from co-electrospinning solutions of TiO_2_–PVP–PAani and pure PAN in DMF through two syringes onto an aluminum collector plate. After spinning, Ti(O_i_Pr)_4_ hydrolysis was achieved in air, followed by thermal stabilization and carbonization at 1000 °C. Cai et al. prepared a CNT/CNF anode by electrospinning PAN/CNT from DMF, followed by heat pressing and carbonization [203]. They found only few bacteria, used to grow a biofilm, on pure carbon fiber anodes, and a much thicker biofilm on the CNT/CNF anode, which was attributed to the increased roughness and hydrophilicity of the anode due to the introduction of the CNTs. Another material combination was suggested by Jung and Roh who used CNF/polypyrrole (PPy) electrospun nanofiber mats as anodes in microbial fuel cells and found a nearly doubled power density with their optimized anodes in comparison with commercial graphite felt [204]. Massaglia et al. suggested N-doped carbon nanofibers as anodes for microbial fuel cells [205], while Karra et al. investigated activated carbon nanofibers and found them to be superior to anodes prepared from granular activated carbon or carbon cloth [206].

Besides these examples of biotechnological fuel cells, diverse others were shown to be producible with electrospun anodes. Working with urea-contaminated wastewater, Barakat et al. suggested carbon nanofibers with embedded NiSn nanoparticles as anodes in direct urea fuel cells [207]. They showed that adding tin as a co-catalyst to nickel, a high current density for urea oxidation could be achieved, and found an average carbonization temperature of 850 °C to be advantages as compared to higher temperatures where the graphite content decreased, leading to a decrease in the catalytic activity. Mohamed et al. used a glassy-carbon electrode coated with electrospun Ni/Pd–C nanofibers as the anode for urea fuel cells, in which the nanofibers were prepared by electrospinning PVA with nickel (II) acetate tetrahydrate and palladium (II) acetate, followed by calcination at a temperature of 900 °C in argon atmosphere [208]. Ni/Cd-decorated electrospun carbon nanofibers were investigated as anodes of urea fuel cells by Abdelkareem, who found a significantly improved electrocatalytic activity for urea oxidation, as compared to anodes without Cd [209].

Perovskites were already described before as possible cathodes for solid oxide fuel cells. Similarly, they can be used for the anodes in these cells. Hu et al. recently described La_x_Sr_1−x_TiO_3_-Gd_y_Ce_1−y_O_2−δ_ electrospun nanofiber mats as possible composite anodes for these cells [210]. These nanofiber mats were prepared by dissolving PVP in DMF, adding lanthanum nitrate, strontium nitrate, and tetrabutyl titanate in different molar ratios, needle-based electrospinning the solution and calcinating it at 900 °C, before the nanofibers were partly grinded to obtain nanoparticles. These nanofibers and nanoparticles were mixed with terpineol solution and in this form coated on both sides of the electrolyte wafers to form cathode and anode, before they were impregnated with Gd_y_Ce_1−y_O_2−δ_. In this way, a La doping ratio of 0.4 was found to be optimal. Addition of Gd_y_Ce_1−y_O_2−δ_ was shown to significantly increase the electrochemical performance, with an optimum Gd doping ration of 0.2. Besides, nanoparticle-based anodes showed a better electrochemical performance than nanofiber-based anodes if only La_x_Sr_1−x_TiO_3_ was used, while this this was reversed for the Gd_y_Ce_1−y_O_2−δ_ impregnated electrodes [210]. The optimized electrodes were afterwards tested in H_2_ and CH_4_ fuel gases where they showed good thermal and redox cycling stability [211]. Besides this type of composite [212,213], other material systems as anodes for solid oxide fuel cells are, e.g., Ni-coated yttria-stabilized zirconia nanofiber mats [21,22,23,24,25,26,27,28,29,30,31,32,33,34,35,36,37,38,39,40,41,42,43,44,45,46,47,48,49,50,51,52,53,54,55,56,57,58,59,60,61,62,63,64,65,66,67,68,69,70,71,72,73,74,75,76,77,78,79,80,81,82,83,84,85,86,87,88,89,90,91,92,93,94,95,96,97,98,99,100,101,102,103,104,105,106,107,108,109,110,111,112,113,114,115,116,117,118,119,120,121,122,123,124,125,126,127,128,129,130,131,132,133,134,135,136,137,138,139,140,141,142,143,144,145,146,147,148,149,150,151,152,153,154,155,156,157,158,159,160,161,162,163,164,165,166,167,168,169,170,171,172,173,174,175,176,177,178,179,180,181,182,183,184,185,186,187,188,189,190,191,192,193,194,195,196,197,198,199,200,201,202,203,204,205,206,207,208,209,210,211,212,213,214,215,216], SrCe_0.8_Y_0.2_O_3−δ_-Ni nanofiber mats [217], and Sr_2_FeTiO_6−*δ*_ nanofiber mats [218].

**Figure 8:**
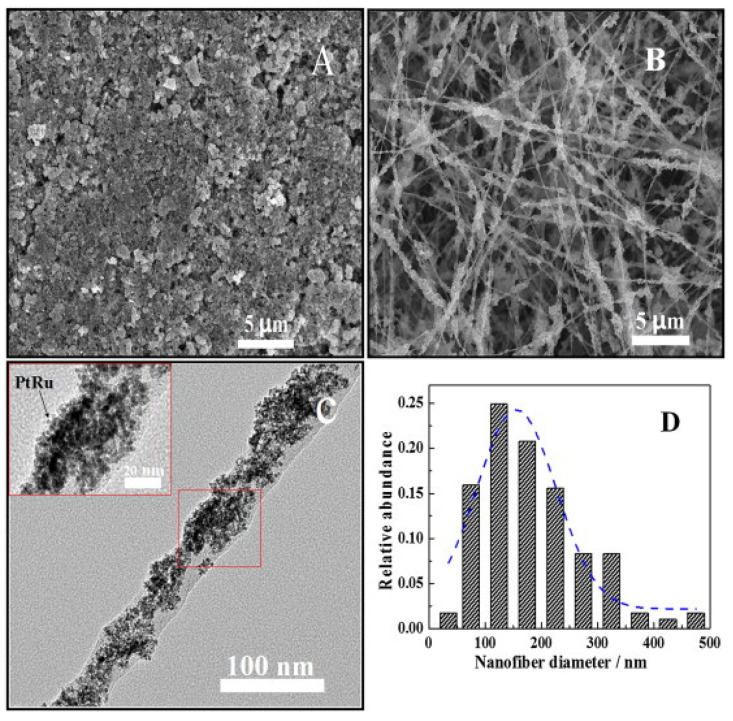
Scanning electron microscopy images of (**A**) a conventional gas diffusion electrode; (**B**) an electrospun gas diffusion electrode; (**C**) transmission electron microscope image of electrospun PtRu-C/Nafion/PVA nanofiber; (**D**) diameter distribution of the electrospun nanofiber mat. Reprinted from [219], with permission from Elsevier.

For direct methanol fuel cells, Chen et al. suggested electrospinning a 3D anodic catalytic layer (Figure 8) to improve catalyst utilization and to reduce charge transfer resistance, in this way significantly increasing the electrochemical performance at simultaneously reduced platinum loading of the electrode [219]. Carbon–CeO_2_ composite nanofiber mats were electrospun by Feng et al. who suggested them as a support for a PtRu anode catalyst in direct methanol fuel cells [220]. Thamer et al. used Ni/C nanofibers mixed with Nafion solution as a catalyst layer on glassy carbon anodes and found high electrocatalytic activity in methanol oxidation which was enhanced by nitrogen doping, while the latter also improved the stability of the catalyst [221]. Hanifah et al. suggested electrospun PVDF/Pt-Pd/RGO-CeO_2_ nanocomposite nanofibers as anode catalyst in direct methanol fuel cells [222]. These fibers were prepared from a solution containing graphite oxide, PdCl_2_ and H_2_PtCl_6_·6H_2_O, mixed with formic acid and Ce(NO_3_)_3_·6H_2_O to reach Pt-Pd/RGO-CeO_2_. This was added to a PVDF solution in N-Methyl-2-Pyrrolidone to allow for needle-based electrospinning. In this way, Pt-Pd/RGO-CeO_2_ nanocomposites in a PVDF nanofiber matrix could be prepared which could be used as catalyst nanofibers for direct methanol fuel cell anodes. Other materials used to prepare the anodes of direct methanol fuel cells are, e.g., TiO_2_/C with platinum and ruthenium catalyst [223,224], PPy nanofiber networks [225], and CeO_2_-C nanofibers decorated with Pt-Co nanoparticles [226].

Besides these types of fuel cells, electrospun nanofiber mats are only scarcely applied as parts of anodes, e.g., Pt/SnO_2_ nanofibers as electrocatalyst in polymer electrolyte membrane fuel cells to support hydrogen oxidation reaction and block oxygen reduction reaction there [227]. Another often reported application of electrospun nanofiber mats is, due to their tailorable porosity, the membrane of different fuel cells.

### 5.3. Electrospun Membranes

In H_2_/Br_2_ regenerative fuel cells, Park et al. applied Nafion perfluorosulfonic acid/PVDF electrospun membranes, containing 2–5 nm fine fibril strands from Nafion and PVDF aligned to the fiber axis [215]. The membranes were produced by hot-pressing and subsequent thermal annealing. With increasing PVDF content, a decrease in proton conductivity, water/electrolyte swelling and permeability for Br_2_/Br_3_^−^ was found, making a membrane with 20% PVDF suitable for H_2_Br_2_ fuel cells and showing nearly 50% higher power output than with a common Nafion 212 membrane [228]. Nafion/polyphenylsulfone nanofiber mats were produced by simultaneous electrospinning of both components and showed good water swelling and mechanical performance as well as proton conductivity [229]. Many other nanofiber mats based on material blends including Nafion are reported in the scientific literature [230,231,232,233,234].

Besides these material combinations, several others are suggested for different fuel cells. Bipolar membranes can be used, e.g., for self-humidifying H_2_/air fuel cells, besides other electrochemical devices [235]. Combining a metal-organic framework (MOF) with sulfonated poly(phthalazinone ether sulfone ketone) (SPPESK) was suggested by Wu et al. as a possible membrane for high proton conductivity in proton exchange membrane fuel cells, working at high temperatures and under anhydrous conditions [236]. The nanofibers were highly oriented and showed thus high proton conductivity, combined with oxidative stability and resistance of methanol permeability, making them well suitable for direct methanol fuel cells. Similarly, Gong et al. suggested ordered SPPESK nanofiber mats to reach high tensile strength and cell power density [237]. Another high-temperature membrane was prepared by Muthuraja et al. who used poly(aryl sulfone ether benzimidazole) membranes for proton exchange fuel cells [238]. Electrospinning this material from dimethyl sulfoxide (FDMSO) in a needle-based process, they found high proton conductivity and oxidative stability due to the sulfone and ether links in the polymeric backbones as well as a highly porous structure, enabling high acid doping and increasing proton conductivity. Using highly oriented sulfonated PI nanofibers, Tamura and Kawakami produced proton exchange membranes for fuel cells with high chemical and mechanical stability as well as high proton conductivity parallel to the nanofibers [239]. Electrospun nanofibers mats with pore size gradients were suggested by Balakrishan et al. for polymer-electrolyte membrane fuel cells [240], who also studied degradation of such electrospun gas diffusion layers [241], while Kallem et al. highlight some possible strategies for nanofiber-based proton exchange membranes with aligned nanofiber mats [242].

**Figure 9:**
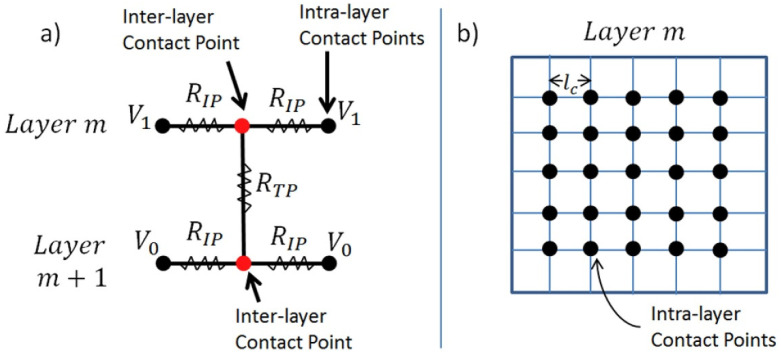
Resistor network of a nanofiber mat: (**a**) conductivity between layers; (**b**) formation of contact points inside a single layer. Reprinted from [243], with permission from Elsevier.

Finally, a theoretical approach should be mentioned. DeGostin et al. developed a fiber network model to predict the conductivity of electrospun nanofiber mats [243]. They modeled the 3D nanofibrous morphology to approximate fiber layering and membrane swelling in water. By translating the fiber network into a resistor network, as shown in Figure 9, they found similar conductivity as experimentally measured in electrospun proton and anion exchange membranes in fuel cells.

In all aforementioned applications in the research area or electrospun elements for fuel cells, the fiber and mat morphologies are again of high interest. Most of the aforementioned papers investigate these properties by SEM, partly by TEM, and aim at providing porous nanofiber structures to further improve the SSA of the corresponding nanofiber mats.

Generally, electrospun nanofiber mats can be applied as cathodes, anodes, or membranes of fuel cells. The possibilities to tailor the morphology of nanofibers and mats, in this way creating the desired nano- and micropores to reach a high SSA, and the physical and chemical material properties, e.g., by varying the carbonization/calcination process, offer a broad range of available properties which can be optimized towards the respective applications.

## 6. Supercapacitors

**Figure 10:**
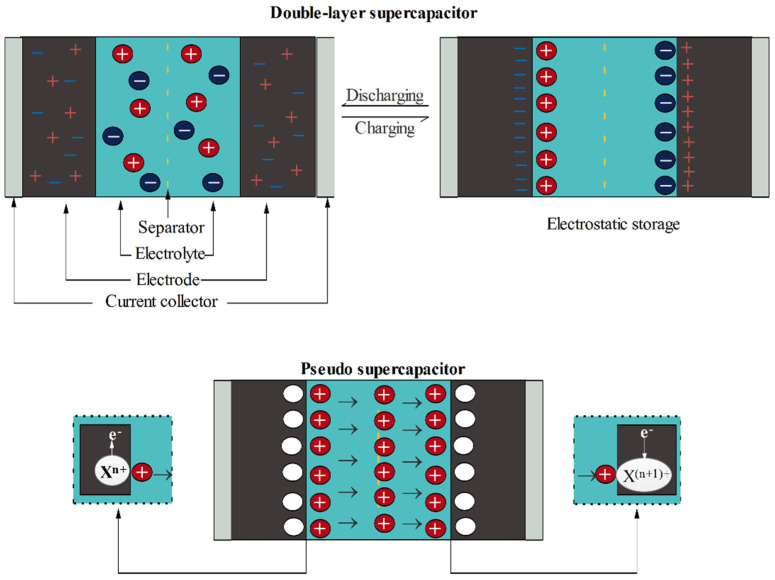
Schematic illustration of various supercapacitor types.

Supercapacitors, also called electrochemical capacitor, double layer capacitors and ultra-capacitors, are considered as one of the novel electrical energy storage methods. Compared to batteries, they deliver higher power rates and life times. However, they suffer from lower stored energy than batteries. So, their practical usage is limited by their poor energy density (4–5 Wh·kg^−1^), whilst batteries display energy output in the range of 100 to 200 Wh·kg^−1^. Supercapacitors could be applied in different electronic equipment, including hybrid vehicles, portable devices, and many more. They are mainly comprised of active and inert components. Electrode and electrolyte are classified as the active materials, whilst current collector, binder, and separator are categorized as the inactive ones [175,244]. Figure 10 schematically illustrates the basic structure of various supercapacitors.

### Electrospun Fibers as Supercapacitor Electrode Materials

Based upon the operational mechanism, supercapacitors can be classified into electrochemical double layer capacitance (EDLCs) and pseudo capacitance classes. In the EDLC category, physical accumulation of the charge carriers and ions on the electrode-electrolyte interfacial layer results in the energy storage. Carbon-based materials such as carbon aerogel, graphene, CNT, and many more belong to the EDLC materials, whereas metal oxides and conducting polymers have shown pseudo capacitance behavior as they store energy through physio-chemical reactions [245,246].

Carbon-based materials are frequently described for fabrication of the EDLC electrodes. These materials have revealed numerous advantages such as great electrical conductivity, proper specific surface area, appropriate chemical stability, low cost, and so on. According to their operating mechanism, surface area and morphology parameters influence the capacitor performance. In addition, existence of the functional groups on the carbon-based materials increases the ion adsorption, stemming from the wettability enhancement. Therefore, numerous researches have illustrated that optimized pore size and surface area factors as well as the existence of functional groups on the material surface could lead to performance enhancement of the carbon-based electrodes. However, contact resistance of the carbon particles is a serious weakness associated with the carbon-based materials. The reason is attributed to increment of the electrode resistance and therefore reduction of the capacitance efficiency [245,246].

**Figure 11:**
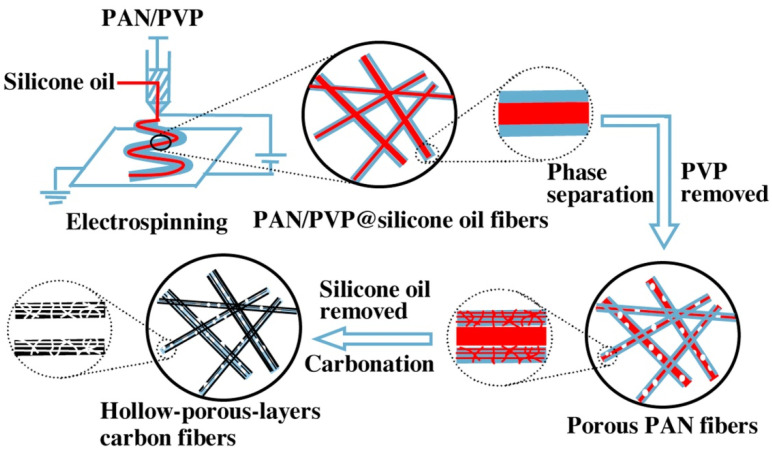
Preparation steps of hollow-porous multilayered ultrafine carbon fibers. Reprinted from [247], with permission from Elsevier.

Recent evidences suggest that carbon-based nanofibrous materials can enhance ion migration into the active surfaces and improve interfacial charge transportation. This could be linked with the highly porous structure of the nanofibrous materials and their great electrical conductivity. As an example, carbon nanofibers (CNFs) display high electrical and thermal conductivities as well as excellent mechanical and chemical stabilities. Commonly, CNFs are fabricated through electrospinning of a host polymer followed by a carbonization procedure at high temperatures. PAN, PVP, and PI belong to the notable host polymers. Moreover, several guest polymers such as PMMA and polystyrene (PS) could be applied to fabricate porous CNFs. The porous CNF structures are produced as a result of guest polymer removing after the heat treatment step [245,246]. Figure 11 displays the applied procedure for fabrication of hollow-porous CNF by He et al. [247]. It is worth noting that increasing the calcination temperature leads to a reduction of the obtained carbon fiber diameter and results in superior electrochemical behavior as it is declared by Pech and Maensiri [248]. Several attempts applied for synthesis of the CNF structures by using the electrospinning procedure are listed in Table 5.

High electrical conductivity, wide operational potential window, and low cost belong to the main advantages of conducting polymers. In such electrode materials, energy is stored through oxidation or reduction reactions. PAni, PPy, poly(3,4-ethylenedioxythiophene) (PEDOT), polyindophenine, and p-phenylenevinylene (PPV) are common examples of conducting polymers. However, poor cycling performance and mechanical degradation are the major drawbacks associated with these electrode materials. The reason may be linked with the surface area reduction and thus specific capacitance decrement by increment of the mass loading. Therefore, porous conducting polymers with improved specific surface area could be great candidates for being applied in such electrochemical devices. Obtained data from several researches has illustrated that the nano-sized conducting polymers could reveal boosted electrochemical behaviors caused by their highly porous structures and superior surface-to-volume ratios. In fact, ion diffusion into the bulk of the electrode material is accelerated in the nanostructured conductive polymers as a result of stronger participation of the applied materials in the redox reactions [258,259]. Chaudhari et al. demonstrated specific capacitance of 267 F·g^−1^ at a current density of 0.35 A·g^−1^ for the electrospun PAni fibers. However, the synthesized PAni particles showed lower specific capacitance of 208 F·g^−1^ [260]. In addition, Maio et al. reported specific capacitance of 601 F·g^−1^ at a current density of 1 A·g^−1^ for the fabricated hollow PAni nanofibers [261]. Sahoo et al. reported the highest specific capacitance of 284 F·g^−1^ at a current density of 1 A·g^−1^ for the PPy nanofibers [262]. Moreover, Liu et al. presented a specific capacitance of 463 F·g^−1^ for the synthesized Ppy nanotubes at a current density of 0.3 A·g^−1^, while the electrospun PAni exhibited much lower specific capacitance of 243 F·g^−1^ at similar current density [263]. Furthermore, the enhanced electrochemical performance of other electrospun conductive polymers such as PPV [264], polyindophenine [265], and PEDOT [266] has been widely reported.

Compared with the carbon-based materials and conducting polymers, metal oxides have illustrated superior energy storage efficiency, along with higher cycling stability. These materials are able to store energy through both physical accumulation and redox reaction. RuO_2_, MnO_2_, NiO, and CoO_x_ are of the widely studied electrode metal oxides. However, several challenges such as low electrical conductivity and high cost have restricted their development. As claimed by numerous researchers, the mentioned drawbacks could be addressed through synthesis of the nano-sized transition metal oxides [267,268]. For example, Hyun et al. displayed high specific capacitance of 889 F·g^−1^ and 30% capacity loss after 2000 cycles for the fabricated RuO_2_ nanofibers [269]. Kolathodi et al. reported higher ionic and electrical kinetics as well as improved capacity retention for the MnO_2_ transition metal oxide in the nanofiber formation due to higher surface-to-volume ratio [270]. NiO nanofibers also exhibited a specific capacitance of 182 F·g^−1^ at a current density of 2 A·g^−1^ and maintained 98.2% of their capacity after 5000 cycles [271]. In addition, high specific capacitance of 700 F·g^−1^ and great cycling stability of 96% were illustrated by applying electrospun hollow NiO fibers as the supercapacitor electrode [272]. Moreover, electrospun Co_3_O_4_ fibers have shown a specific capacitance of 407 F·g^−1^ and retained 94% of their capacitance after 1000 cycles [273].

It is extensively reported that graphene has the highest theoretical specific capacity in comparison with the other carbon-based materials. Proper chemical stability, high surface area, and low cost are some appealing characteristics of graphene-based electrodes, while restacking of the graphene layers has blocked approaching to the reported theoretical capacity value. In addition, the evaluated single-phased nanofibrous materials have not been ideal for being applied in the practical usages. As an example, limited specific capacity and low energy density are some of poor properties of nano-structured EDLC materials. In the case of pseudocapacitive nanofibers, poor electrical conductivity and low cycle stability are identified as the main disadvantages. So, nanocomposite materials, composed of several electrode materials, such as carbon-based structures, metal oxides, conducting polymers, etc., have been widely developed to enhance electrochemical performance of the electrodes [245,246]. To date, numerous studies have been devoted to figure out various electrochemical characteristics of the electrospun nanocomposites. For instance, electrospinning of the combination of PAni with various carbon-based materials including CNT [274,275], graphene [276,277], and CNFs [278,279] has resulted in the improved specific capacity as well as enriched cycling durability. Table 6 summarizes recent attempts for synthesis of electrospun nanocomposite materials as supercapacitor electrodes.

To date, supercapacitors have been recognized as high power density energy storage devices. Nevertheless, synthesis of appropriate electrodes for commercialization of supercapacitors has remained as a challenge. Electrospun structures have widely shown their great potentials as electrode materials of supercapacitors due to providing higher conductivity as well as appropriate structural stability and great porosity. Despite numerous successful efforts in the field of electrospun supercapacitor fabrication, there are several downsides (e.g., nonsufficient electrical conductivity) that must be addressed in the future. Evaluation of selenides/CNF and tellurides/CNF composites for conductivity enhancement, synthesis of metal oxides with complex interior for improvement of electrochemical behavior, and synthesis of one-dimensional porous electrospun fibers for boosting the electrical conductivity could be valuable explorations for elimination of drawbacks associated with supercapacitors.

## 7. Electrochemical Solar Cells

**Figure 12:**
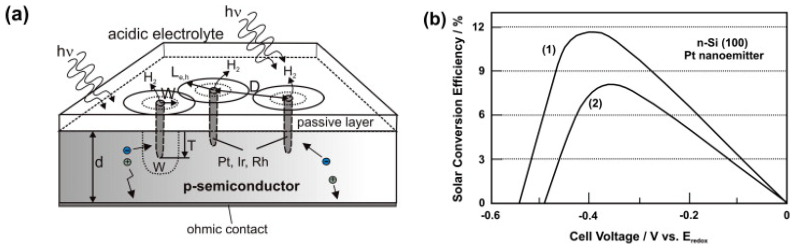
(**a**) Electrochemical solar cell design for protruding emitter noble metals in the electrolyte; (**b**) exemplary solar conversion efficiency at the redox electrolyte contact under an illumination of 100 mW cm^−2^ after different pre-treatments: (1) after photocurrent oscillation in 0.1 M NH_4_F followed by electrochemical pore deepening in 1 M NaOH; and (2) after emersion at the decreasing branch of a photocurrent oscillation only. Reprinted from [300], with permission from Elsevier.

Remembering the definition from the abstract section that electrochemical devices convert chemical reactions into electrical energy or vice versa, actually all solar cells could be described as electrochemical solar cells. Indeed, there are several different definitions of electrochemical solar cells. Most often, however, solar cells are differentiated into solid state solar cells [295,296] and photoelectrochemical solar cells in which chemical reaction with ions or water take place [297,298]. In the latter, the contact potential between a semiconductor and an electrolyte leads to the separation of charge carriers which were photoinduced [299,300], or in other words, the potential barrier which is necessary for charge separation in solar cells is realized here by the semiconductor-electrolyte junction [301]. The principle of such cells is depicted in Figure 12 [302].

Recently, dye-sensitized solar cells (DSSCs) and perovskite solar cells belong to the often investigated so-called third-generation photovoltaic cells which can be described as electrochemical solar cells. This is why diverse review papers are available, giving a good overview of DSSCs [302,303,304,305,306] as well as perovskite solar cells [307,308,309,310,311]. Nevertheless, research on other electrochemical solar cells and specialized devices is still going on.

Vijayaraghavan et al., for example, used spray pyrolysis to deposit CdTe thin films on a TiO_2_ nanoparticle layer as photo-active semiconductor in combination with iodine/triiodide electrolyte and found an efficiency of 0.4% for an optimized CdTe layer thickness [312]. A CdSeS composite film was applied by Hazra et al. in their electrochemical cell, leading to reduced photo degradation which is a severe problem in electrochemical cells [313]. The ternary alloy Cd_1−x_Zn_x_Se was suggested by Kissinger to prepare an electrochemical solar cell with Na_2_S-S-NaOH as redox electrolyte, reaching efficiencies of up to 4.5% [314]. Another often reported material is WSe_2_ which was shown to have high efficiencies of about 14–17% [315,316,317].

Aljafari et al. suggested combining an electrochemical solar cell with a supercapacitor into a single device, including a PVA/hydrochloric acid-based gel electrolyte, multi-walled CNT, and fluorine-doped tin oxide as counter and working electrodes, respectively, where the working electrode consists of a composite of a conducting polymer and synthetic dyes, such as methylene blue, methyl orange, or Prussian blue [318]. They reported strong photo-electrochemical reaction especially for methylene blue. In the aforementioned papers, crystallinity of the active components is regarded as more important than the surface morphology.

Besides these and a few other reports found in the recent scientific literature, electrochemical solar cells mostly refer to DSSCs for which the reader is referred to the aforementioned or other specialized review papers.

## 8. Sensors

**Figure 13:**
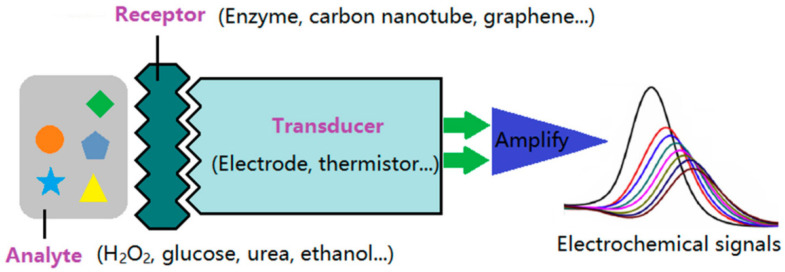
Schematic diagram of an electrochemical sensor. Reproduced from [325], originally published under a CC-BY license.

Sensors are devices required for authentic detections in various fields of chemical analysis, food assessment, clinical diagnosis, and many more. They have been developed based upon several detection technologies including fluoro-photometery, chemoluminescence, liquid chromatography, spectrophotometry, and electrochemistry [319,320]. Among them, electrochemical sensors received tremendous attention resulting from their wide detection range as well as high selectivity [321]. This kind of sensors was first introduced by Clark in 1962 [322]. They are commonly comprised of a receptor and a signal transducer (Figure 13). In such devices, the interactions between the sensitive receptor and analyte are measured. Then, an electrical signal is applied to clarify the level of interaction. Through analysis of the reported signal, information about the material content could be obtained.

According to the mentioned operational mechanism, such sensors mainly contain an electrode connected to a sensitive component. Materials with specific performance are deposited on the electrode surface. By applying an external voltage, the utilized specific materials take part in a redox reaction which results in generation of a current. Then, the produced current is transferred to a signal analysis system to report the binding efficiency. An ideal electrochemical sensor should reveal low detection limit and appropriate selectivity. In addition, it is vital to report the result in a short response time [321].

Electrospun membranes are great candidates to design highly efficient electrochemical devices, especially electrochemical sensors. This could be attributed to the unique characteristics of the nanofibrous mats, such as high SSA, proper porosity structure, tiny pores, interconnected fibers, etc. In recent decades, great performance features of different electrospun electrochemical sensors have been extensively reported. The evaluated nanofibrous structures have been mainly obtained though electrospinning of various kinds of polymeric, carbon-based, and metal oxide materials [323,324].

### 8.1. Electrochemical Sensors Based on Electrospun Polymeric Fibers

Enzymes are able to catalyze numerous interactions due to their catalytic activity characteristics. They are widely utilized on the electrode surfaces for sensing activity regarding to their proper sensitivity along with great selectivity. Notably, stabilizing of the enzymes on the surface of the electrodes is considered as a critical step in sensor fabrication procedure. Physical adsorption, sol–gel, and self-assembly are some of the commonly applied routes for enzyme stabilization. Meanwhile, unique characteristics of the polymeric nanofibers have facilitated immobilization of the enzymes on the surface of the electrodes. This could be carried out through direct and indirect methods. In the prior technique, enzymes are embedded into the polymeric nanofibers by direct loading of enzymes into the electrospinning solutions, whereas enzymes could be added to the electrospun polymeric nanofibers by using the post-modification processes [324,325]. As an example, Ren et al. immobilized glucose oxidase (GOx) by direct electrospinning of PVA/GOx on the surface of an electrode followed by a cross-linking procedure. The as-spun structure showed linear response in the range from 1 to 10 mM and detection limit of 0.05 mM [326]. In another attempt, Arecchi et al. displayed linear response range of 1 to 10 mM and detection limit of 6 µM by covalent stabilization of GOx on a Nylon-6 electrospun membrane [327]. Several recent approaches focusing on modification of the electrodes by using the electrospun polymeric fibers are summarized in Table 7.

### 8.2. Electrochemical Sensors Based on Carbon Nanofibers

Wide potential window, cost effectiveness, and inertness are well-known intrinsic features of the carbon-based electrodes. Carbon materials have been mainly applied in various forms including carbon fibers, carbon powder, graphite, and many more. With development of nano-sized materials, CNFs have been applied in fabrication of several electrochemical applications, specifically sensing and electro-analysis. CNFs are introduced as an efficient matrix for immobilization of enzymes due to several advantages such as excellent mechanical strength, appropriate conductivity, and high SSA. In addition to their performance as ideal matrixes, proper electrical conductivity has provided their potential for acting as transducers. This may be attributed to the existence of more edge planes in the CNF structure [334]. CNF-based electrochemical sensors are commonly fabricated using an electrospinning method followed by a carbonization technique. Wu et al. investigated catalytic activity of an electrochemical glucose sensor designed by CNFs which resulted in linear response range of 0.1 to 78 µM and low detection limit of 0.07 µM [335]. Based on a study performed by Bae et al., increasing the porosity, crystallinity, and orientation of the carbon nanofibers could result in higher current and superior sensitivity [336]. In addition, Liu et al. reported linear range of 1 to 800 µM with a low detection limit of 0.6 µM for a H_2_O_2_ electrochemical sensor for an electrode loaded by Pt nanoparticles and CNFs [337]. A sensitivity down to 1 nM and high selectivity for glucose molecules was presented by Kim et al., using cobalt-oxide-incorporated multichannel carbon nanotubes [338], while Simsek et al. reached a detection limit of 0.3 µM glucose with Ni nanoparticles adhered to a CNF network [339].

A summary of the most recent fabricated sensors based on CNFs is provided in Table 8.

### 8.3. Electrochemical Sensors Based on Metal and Metal Oxide Nanofibers

Distinctive characteristics of the electrospun metals and metal oxides, such as high specific surface area, interconnected pores, proper porosity, fast response, high sensitivity, etc., have made them a great candidate for fabrication of various electrospun sensors. Nanofibrous metal oxides are commonly produced through the following procedures: (a) electrospinning of polymer solution embedded with metal oxide precursor, and (b) dipping the electrospun fibers into a metal oxide precursor solution. Both methods are followed by a calcination step. During the calcination process, the utilized polymer content is degraded, while metal-oxide crystals are grown and nucleated by temperature increment. Numerous literatures have reported excellent performance of the mentioned electrospun fibers for sensing of different targets, including glucose, ascorbic acid, cholesterol, uric acid, and so on. These kinds of electrospun fibers are applied in synthesis of enzyme and none-enzyme electrochemical sensors. The non-enzyme sensors act based on catalyzed reactions. So, electro-catalytic materials (e.g., metals and metal oxides) play a key role in providing the aforementioned reactions. The non-enzyme electrochemical sensors could reveal superior cycling stability compared with the enzyme ones. In addition, they are synthesized through easier processes as they do not require an immobilization procedure. Notably, the combination of nano-dimensional metals and metal oxides with carbon-based materials has been broadly applied to enhance the performance of signal transduction in the non-enzyme electrochemical sensors. Nano-sized metals and metal oxides display significantly superior transducer activity as compared to the micro-sized ones [349,350,351]. Table 9 lists several recent studies which have explored electrochemical behavior of electrospun sensors through fabrication of metal and metal oxide nanofibers and their combination with CNFs.

As a working principal, effective sensing and conversion of the signals are the main features of an ideal electrochemical sensor. In addition, efficiency, size, and price are other critical issues related to an appropriate electrochemical sensor. Applying nanotechnology for the fabrication of electrochemical sensors has led to production of more efficient, smaller, and cheaper electrochemical sensors due to high specific surface area, great electrical conductivity, and so on. Nevertheless, progress and development of technology have a greater demand for increment of sensitivity and specificity. Among various materials applied for synthesis of electrospun non-enzyme sensors, fibrous composites of metal or metal oxides with carbon nanofibers have illustrated desirable features and could be the future trend of most researches. In addition, production of multi-analysis electrochemical sensor systems is of interest to researchers and developers. Any advancement in this area could be beneficial for various medical fields.

## 9. Conclusions

In this review, the broad range of recent approaches on applications of electrospun fibers in various electrochemical structures has been evaluated. Based on the investigated efforts, electrospun fibers have enhanced various characteristics of electrolytic cells, battery structures, fuel cells, supercapacitors, solar cells, and sensors. The great potential of such structures for being applied as different components of the mentioned cells has been widely illustrated. Regarding the possible materials for nanofiber mats in electrochemical devices, a broad range of materials can be found in the recent literature, from quite common, easily spinnable ones like PAN along carbon nanofibers, e.g., produced by stabilizing and carbonizing PAN or other precursor fibers, to semiconducting or metallic nanoparticles integrated in a polymer matrix or as pure nanofibers produced by calcination of the electrospun composite fibers. While electrode materials need to be conductive and are thus often produced from carbon nanofibers, partly also from conductive polymers, the mechanical properties and pore sizes are more important for separating membranes which thus often consist of PAN and other reliably spinnable materials. Sensors, on the other hand, necessitate specific metallic or other constituents to reach a high sensitivity towards a desired molecule.

Overall, nanofibrous structures have promising industrial applications in various electrochemical cells regarding their unique and fabulous features. Nevertheless, there is still a lot of challenges to be solved and open questions to be investigated. For electrolytic cells, e.g., research should be broadened beyond electrolytic water splitting and degradation of dyes and other contaminants. Regarding batteries, the main goals are a high efficiency and improved functionality, necessitating further improvements of all battery components, especially in terms of mechanical stability, electrical conductivity, and ionic conductivity. For the diverse kinds of fuel cells in which electrospun nanofiber mats can be applied, the most crucial parameters for the electrodes are specific surface area and conductivity as well as introducing a suitable catalyst, while for membranes, the pore size distribution and possible selectivity governs the choice of materials and structures. In supercapacitors, high conductivity, structural stability, and great porosity of nanofiber mats are again the most important parameters. Especially the electrical conductivity and the electrochemical properties need to be improved further, which may be done by introducing composite materials like selenide/CNF or telluride/CNF nanofibers or metal oxides with complex interior, respectively. Research on electrochemical solar cells is mainly related to DSSCs or perovskite solar cells recently; here, finding non-toxic, abundantly available materials with high efficiency as dyes or for other parts of these solar cells would highly increase the further interest in this topic. Finally, nanofiber mats for applications in electrochemical sensors need further increased sensitivity and specificity, ideally in the form of multi-analysis sensor systems. We hope that our review paper can support future research in all these areas.

## Figures and Tables

**Table 1:** The most recent approaches toward fabrication of nanofibrous cathodes.

Material	Capacity (mAh·g^−1^)	Cycling Stability	Autor (Year)	Ref.
Li_2_CoTi_3_O_8_/TiO_2_	82 at 0.1 C	83% after 25 cycles	Kap et al. (2020)	[99]
LiFePO_4_ nanocrystals/carbon nanofibers (CNFs)	152 at 0.5 C	98.2% after 500 cycles	Cao et al. (2020)	[100]
V_2_O_5_/GO	342 at 0.5 C	80% after 20 cycles	Ahmadian et al. (2020)	[101]
Li_2_MnTiO_4+*z*_	210 at 0.1 C	95.3% after 100 cycles	Vu et al. (2020)	[102]
LiFe_0.8_Mn_0.2_PO_4_/C	169.9 at 0.1 C	160 after 200 cycles	Chen et al. (2020)	[103]
LiFe_0.4_Mn_0.6_PO_4_/CNFs	133.5 at 1 C	138.8 after 100 cycles	Yang et al. (2020)	[104]

**Table 2:** Summarization of some of the most recent approaches toward fabrication of nanofibrous anodes.

Material	Energy Storage Mechanism	Capacity (mAh·g^−1^)	Autor (Year)	Ref.
CNF	Intercalation	294 at 0.2 A·g^−1^	Li et al. (2020)	[118]
MnCo_2_O_4_	Conversion reaction	701 at 0.5 A·g^−1^	Zhu et al. (2020)	[119]
TiO_2_/CNF		399	Su et al. (2020)	[120]
Fe_3_O_4_/CNF		1635 at 1 A·g^−1^	Liu et al. (2020)	[121]
Sn_4_P_3_/CNF		710	Ran et al. (2020)	[122]
P/CNF	Alloying reaction	730 at 0.1 A·g^−1^	Liberale et al. (2020)	[123]
Si/PCNF		1033 at 5 A·g^−1^	Tian et al. (2020)	[124]
SnP_0.94_/CNF	Conversion/Alloying reactions	750 at 0.1 A·g^−1^	Yadav et al. (2020)	[125]
SnSe/CNF		405 at 1 A·g^−1^	Xia et al. (2020)	[126]
SnSe/N-doped CNF		460 at 0.2 A·g^−1^	Shaji et al. (2020)	[127]

**Table 3:** Recent approaches toward fabrication of electrospun separator applicable in Li-ion batteries.

Material	Porosity (%)	Tensile Strength (MPa)	Electrolyte Uptake (%)	Ionic Conductivity (mS·cm^−1^)	Autor (Year)	Ref.
PAN	67.7	11.3	478.2	1.97	Dong et al. (2020)	[151]
PAN/PBS	59.3	7.66	665	2.1	Wei et al. (2020)	[152]
PVA/ZrO_2_	78	14.5	350	2.19	Xiao et al. (2020)	[153]
PI/Al_2_O_3_	81	-	912	-	Iaritphun et al. (2020)	[154]
PVDF-HFP/SiO_2_	89.7	5	483	-	Xu et al. (2020)	[155]
PVDF-HFP/PI	85.9	9.76	483.5	1.78	Cai et al. (2020)	[156]
PVDF-HFP/LAGP	-	-	215	3.18	Liang et al. (2021)	[157]
PVDF/TPP/CA	90	6.9	301	4.4	Chen et al. (2020)	[158]
PAN/HCNFs@PVDF/UiO-66	77.61	24.77	570.97	1.59	Fa et al. (2021)	[159]

**Table 4:** Ionic conductivity of the electrospun and solution-casted membranes with similar chemical compositions.

Material	Fabrication Method	Ionic Conductivity (mS·cm^−1^)	Author (Year)	Ref.
PEO/PC/LiClO_4_	Casting	1.7 × 10^−3^	Banitaba et al. (2019)	[170]
	Electrospinning	5 × 10^−2^		
PEO/Li(TFSI)	Casting	1 × 10^−3^	Walk et al. (2018)	[171]
	Electrospinning	4.4 × 10^−3^		
PEO/EC/LiClO_4_	Casting	8 × 10^−3^	Banitaba et al. (2020)	[169]
	Electrospinning	1.72 × 10^−1^		
PEO/EC/LiClO_4_/Al_2_O_3_	Casting	4.4 × 10^−3^	Banitaba et al. (2019)	[172]
	Electrospinning	5.9 × 10^−2^		

**Table 5:** Electrospun carbon nanofibers as supercapacitor electrode material.

Host [Guest] Components	Thermal Treatment	SSA (m^2^.g^−1^)	Electrochemical Performance			Author (Year)	Ref.
			Specific Capacity (F·g^−1^)	Energy Density (Wh·kg^−1^)	Capacity Retention		
PAN	Stabilized at 280 °C for 1 h, carbonized at 700–800 °C, and activated by N_2_ and steam for 30 min	1230	173 at 0.01 A·g^−1^	-	-	Kim & Yang (2003)	[249]
Polybenzimidazole	Activated by N_2_ and steam at 750–850 °C for 30 min	1220	178 at 0.007 A	-	-	Kim et al. (2004)	[250]
PVDF/PVP	Dehydrofluorinated at 60 °C for 1 h and heated to 800 °C in N_2_	1084	331 F·g^−1^ at 1 A·g^−1^	13.1	89.2% after 2000 cycles	Ma et al. (2019)	[251]
PAN/PMMA	Stabilized at 250 °C for 4 h and carbonized at 800 °C for 1 h in N_2_	224	210 F·g^−1^ at 1 A·g^−1^	-	100% after 2000 cycles	Lai et al. (2015)	[252]
PAN/PVP	Stabilized at 300 °C for 2 h, carbonized at 300 to 970 °C for 3 h in N_2_, and activated by CO_2_ at 850 °C for 1.5 h	531	220 F·g^−1^	-	-	Niu et al. (2011)	[253]
Polyamic acid (PAA)/PVP	Stabilized at 280 °C for 2 h, carbonized at 280 to 900 °C for 7 h in Ar, and activated by KOH at 850 °C for 2 h	743.5	211.7 F·g^−1^	23.1	-	He et al. (2020)	[254]
poly(styrene-co-acrylonitrile)/PAN/PVP	Stabilized at 250 °C for 2 h and carbonized at 800 °C for 1 h in N_2_	26	239 F·g^−1^ at 1 A·g^−1^	15	92.33% after 10,000 cycles	Kim et al. (2020)	[255]
Lignin/PVA	Stabilized at 250 °C in N_2_ for 2 h and carbonized at 900 °C for 2 h in N_2_	2005	205 F·g^−1^ at 1 A·g^−1^	-	83% after 1500 cycles	Ago et al. (2016)	[256]
PAN [PVP/Silicone oil]	Stabilized at 300 °C for 2 h, carbonized at 300 to 970 °C for 3 h in Ar, and activated by KOH at 850 °C for 1.5 h	1120.3	231.6 F·g^−1^	15.1	99.7% after 2000 cycles	Ishita & Singhal (2020)	[257]
PAN [PS]	Stabilized at 280 °C for 2 h and carbonized at 800 °C for 1 h in N_2_	432	271.6 F·g^−1^ at 0.5 A·g^−1^	18.8	100% after 5000 cycles	Ishita & Singhal (2020)	[257]

**Table 6:** Electrospun composites applied as electrode in supercapacitor devices.

Electrospun Hybrid Materials	Thermal Treatment	Electrochemical Performance			Author (Year)	Ref.
		Specific Capacity (F·g^−1^)	Energy Density (Wh·kg^−1^)	Capacity Retention		
PAni/CNF	Stabilized at 280 °C for 4 h and carbonized at 800 °C in N_2_	439 at 1 mA·cm^−2^	68.6	90% after 5000 cycles	Anand et al. (2020)	[280]
PAni/heteroatom-doped CNF	Annealed at 250 °C for 2 h and pyrolysed at 900 °C for 1 h in N_2_	680.8 at 0.5 A·g^−1^	27.7	93.5% after 3000 cycles	Zhu et al. (2020)	[281]
PAni/MnO_2_/CNF	Stabilized at 280 °C for 2 h and carbonized at 800 °C for 0.5 h in N_2_	937.66 at 1 A·g^−1^	66.12	97.6% after 5000 cycles	Jalil et al. (2020)	[282]
Graphene/CNT/CNF	Stabilized, maintained at 500 °C for 1 h, and kept at 700 °C	218 at 1 A·g^−1^	62.13	94.98% after 10,000 cycles	Kshetri et al. (2020)	[283]
MoS_2_/graphene/CNF	Pretreated at 450 °C for 1.5 h and carbonized at 800 °C for 2 h in H_2_	334 at 0.5 A·g^−1^	-	83.8% after 5000 cycles	Fu et al. (2020)	[284]
Nitrogen-oxygen co-doped CNF	Stabilized at 200 °C for 1 h, annealed at 1000 °C for 0.5 h in N_2_, and maintained at 600 °C for 1 h	320 at 1 A·g^−1^	17.92	94.5% after 5000 cycles	Dai et al. (2020)	[285]
PI/CNF	Solvothermal treatment at 200 °C for 12 h	1139 at 5 A·g^−1^	94	90% after 10000 cycles	Zhang et al. (2020)	[286]
Co_3_O_4_/C/CNF	Stabilized at 250 °C for 4 h and carbonized at 950 °C for 1 h	1632 at 5 A·g^−1^	36.6	82.5% after 7000 cycles	Mukhiya et al. (2020)	[287]
MnO_2_/TiO_2_	Calcinated at 500 °C for 1 h	111.5 at 1 A·g^−1^	62	87.2% after 5000 cycles	Kolathodi et al. (2020)	[288]
MnO_2_/porous CNF	Oxidized at 280 °C for 1 h and carbonized at 280 °C for 1 h in N_2_	228 at 1 A·g^−1^	25.3	94% after 10,000 cycles	Jeong et al. (2020)	[289]
ZnFe_2_O_4_/carbon	Stabilized at 250 °C, carbonized at 600 °C, and annealed at 280 °C	237 at 1 A·g^−1^	-	93.1% after 10,000 cycles	Yang et al. (2020)	[290]
Fe_2_MoC/carbon	Stabilized at 250 °C for 2 h and carbonized at 800 °C for 2 h in Ar	347 at 1 A·g^−1^	14.5	93% after 5000 cycles	Hao et al. (2020)	[291]
PAni/MnO_2_/CNF	stabilized at 280 °C for 5.5 h and carbonized at 700 °C for 2 h	289 at 1 A·g^−1^	119	91% after 1000 cycles	Dirican et al. (2020)	[292]
NiCo_2_S_4_/graphite	Carbonized at 2000 °C	1175.2 at 10 A·g^−1^	52.3	94.7% after 10,000 cycles	He et al. (2020)	[293]
NiCo_2_O_4_/CNF	Carbonized	111 at 1 A·g^−1^	40.3	92% after 5000 cycles	Yang et al. (2020)	[294]

**Table 7:** Polymeric nanofibers utilized for modification of the electrochemical sensors.

Support Materials	Target	Linear Response Range	Detection Limit	Author (Year)	Ref.
PAN/PPy/PPy_3_COOH	Glucose	20 nM−2 μM	2 nM	Sapountzi et al. (2020)	[328]
Cellulose acetate/chitosan	Glucose	5 µM–0.75 mM	4.8 µM	Yezer & Demirkol (2020)	[329]
PAN/montmorillonite	Glucose	1.0 × 10^−5^–2.45 × 10^−3^ M and 2.45 × 10^−3^–15 × 10^−3^ M	2.4 µM	Apetrei & Camurlu (2020)	[330]
Chitosan/GO	Glucose	0.05–20 mM	0.02 mM	Mehdizadeh et al. (2020)	[331]
Chitosan/sodium dodecyl sulfate/hemoglobin	Hydrogen peroxide	3–2940 µM	0.16 µM	Kholosi et al. (2020)	[332]
PVA/chitosan	Urea	0.023–0.23 mM	-	Kutlu et al. (2020)	[333]
PAni/GO	Breast cancer biomarker	10^−15^–10^−7^ M	3.01 × 10^−16^ M	Su et al. (2020)	[120]

**Table 8:** Catalytic activity of the electrochemical sensors based on carbon nanofibers.

Support Materials	Target	Linear Response Range	Detection Limit	Author (Year)	Ref.
CNF	Malachite green	0.1–22.1 µM	0.05 µM	Yang et al. (2020)	[340]
CNF	Tramadol	0.05–100 nM	0.05 nM	Jahromi et al. (2020)	[341]
CNF	Cadmium (II)	2–100 ppb	0.11 ppb	Fakude et al. (2020)	[342]
CNF	Paracetamol	2.0 × 10^−9^–5.0 × 10^−8^ and 1.0 × 10^−7^–2.0 × 10^−6^ M	5.4 × 10^−10^ M	Sasal et al. (2020)	[343]
CNF/GO	Uric acid	100–700 µM	0.14 µA.µM^−1^	Aryal & Jeong et al. (2020)	[344]
CNF/β-cyclodextrin	Ascorbic acid	0.9 µM	100–400 µM	Aryal & Jeong et al. (2020)	[345]
CNF/poly(L-aspartic acid)/nanodiamond particles	L-ascorbic acid	0.2 µM–1.8 mM	0.1 µM	Kacer & Erden (2020)	[346]
CNF/PEDOT	neurotransmitters	0.1–9 µM	0.045 µM	Saunier et al. (2020)	[347]
CNF/zeolitic imidazolate framework-8	dihydroxybenzene isomers	0.06 µM	2-400 µM	Yang et al. (2020)	[348]

**Table 9:** Electrospun electrochemical sensors based on metals and metal oxides.

Materials	Target	Linear Response Range	Detection Limit	Author (Year)	Ref.
CuCr_2_O_4_/CuO	Methotrexate	0.1–300 μM	25 nM	Salandari-Jolge et al. (2020)	[352]
WO_3_	Catechol	1–100 μM	0.52 μM	Veeralingam & Badhulika (2020)	[353]
L-cysteine/ZnO	Lead ion	10–140 µg·Lit^−1^	0.397 µg·L^−1^	Oliviera et al. (2020)	[354]
NiCo_2_S_4_/graphene/CNF	Pyrimethanil	0.06–800 µM	20 nM	He et al. (2020)	[355]
Co_3_O_4_/CNF	Hemoglobin	1–12 mM	0.33 mM	Xie et al. (2020)	[356]
NiMoO_4_/CNF	Glucose	0.0003–4.5 mM	50 nM	Rani et al. (2020)	[357]
Graphene/gold	Glucose	0.5–9 mM	55 µM	Shamsabadi et al. (2020)	[358]
MnO_2_/Co_3_O_4_/CNF	Glucose	<10.2 mM	0.02 µM	Wei et al. (2020)	[359]
Ferric ceria	Uric acid	0.5–500 µM	0.3 µM	Shekh et al. (2020)	[360]
ZnO/CNT	Atrazine	10 zM–1 µM	5.368 zM	Supraja et al. (2020)	[361]
CNF/Co	Hydrogen peroxide	<50 mM	10 µM	Riaz et al. (2020)	[362]
Au/Pt/CNF	Mercury ion	10^−15^–10^−6^ M	3.33 × 10^−16^ M	Xie et al. (2021)	[363]
ZrO_2_/graphene	Osteopontin	0.01 pg·mLit^−1^–2.0 ng·mLit^−1^	4.76 fg·mL^−1^	Zhou et al. (2020)	[364]
TiO_2_/CNT/CNF	Bovine hemoglobin	5–80 mM	1.67 mM	Zhu et al. (2020)	[365]
CoFe_2_O_4_/GO	Rutin	0.001–0.1 nM and from 1.0–100 nM	0.94 pM	Ansari et al. (2020)	[366]
CoFe_2_Se_4_/CNF	Hydroquinone	0.5–200 µM	0.13 µM	Yin et al. (2020)	[367]
	Catechol	0.5–190 µM	0.15 µM		
	Resorcinol	5–350 µM	1.36 µM		

## Data Availability

No data were generated in this study.
